# Transcatheter Arterial Chemoembolization: History for More than 30 Years

**DOI:** 10.5402/2012/480650

**Published:** 2012-08-26

**Authors:** Yong-Song Guan, Qing He, Ming-Quan Wang

**Affiliations:** ^1^Department of Oncology, West China Hospital of Sichuan University, Chengdu 610041, China; ^2^State Key Laboratory of Biotherapy, West China Medical School, Sichuan University, Chengdu 610041, China

## Abstract

Transcatheter arterial chemoembolization (TACE) is a minimally invasive technique to treat liver tumors, particularly hepatocellular carcinoma (HCC). TACE was used in early times to treat liver tumor patients with emergencies caused by symptomatic humoral hypercalcemia and develops gradually from the procedures of diagnostic angiography and transcatheter injection of agents and is in particular performed in the treatment of HCC. Since the beginning of this century, TACE has been used extensively in the palliative treatment of unresectable HCC. In recent years, it is indicated in selected patients with early-stage HCC. This review introduces the evolution of TACE for more than 30 years, its role in comprehensive treatment of HCC, the tendency of its refinement in future, and the combination use of TACE with other local ablative methods for the curative result of HCC.

## 1. Introduction 

The term “transcatheter” denotes “performed through the lumen of a catheter” which is commonly used in interventional radiology with the Seldinger technique [1]. This technique is a procedure to obtain safe access to hollow organs, especially blood vessels. It was developed in 1953 by Dr Sven-Ivar Seldinger (1921–1998), a Swedish radiologist from Mora Municipality, Dalarna County. Embolization is another procedure, nonsurgical, and minimally invasive, through placement of artificial embolus, used to treat a wide variety of conditions affecting different organs of the human body [2]. For tumor therapy, this treatment is used to slow or stop blood supply for reducing the size of the tumor [3, 4]. Transcatheter arterial chemoembolization (TACE) in particular has been performed in the treatment of hepatocellular carcinoma (HCC), the most common malignant tumor of the liver [5]. TACE also has a role in delaying the progression of HCC until a donor liver becomes available [6]. Up to now, TACE has had its history for more than 30 years and progressed greatly during the recent decade. Since the beginning of this century, TACE has been used extensively in the palliative treatment of unresectable HCC. In recent years, it is indicated in selected patients with early-stage HCC.

## 2. Evolution of TACE 

In 1930 Brooks reported embolization of a carotid-cavernous fistula, which could be taken as the earliest concept of therapeutic embolization [7]. Since Seldinger described in 1953 his technique, numerous intravascular procedures have been advocated [8]. Until the early 1970s, many talented ideas derived from Seldinger's technique had been attempted to control gastrointestinal bleeding, such as percutaneous selective angiography and arterial infusion of vasopressin by catheterization [9–11]. At that time, therapeutic embolization had been used percutaneously for treatment of arteriovenous malformations, when Rösch applied selective arterial embolization in 1972 to the gastrointestinal tract for intervention of acute bleeding [12, 13]. In 1972, ligation of the hepatic artery was also reported to treat secondary hepatic tumors followed by infusion of the portal vein with 5-fluorouracil [14]. This is a process of tying off blood vessels to block blood flow to the liver. It was proven safe because liver function was reasonably disturbed in all patients but no one had liver failure. The effects were also encouraging: good relief of abdominal pain, weight gain, reduction in tumor size, and tumor necrosis shown on liver biopsy. Earlier, in 1971, the sequelae of hepatic artery occlusion after hepatic artery catheterization was assessed for 119 successful hepatic catheterizations from January 1963 through February 1969, by which patients with primary or secondary liver tumors were treated with infusion chemotherapy [15]. The catheters had been retained in place for weeks, even more than 10 months, resulting in complete or partial blockage of the hepatic artery in 18 patients. Nevertheless, hepatic artery occlusion was well tolerated in these patients, which challenged the opinion that hepatic artery interruption is lethal and must be avoided which had been established since 1933 and had been quoted for innumerable times [16]. Doctors have come to realize that the liver has such an abundant blood supply as comes from the hepatic artery, the portal vein, and neighboring collateral arteries so that incidence of liver infarction is very rare [17]. 

Tumor embolization, one of the processes of therapeutic embolization, is defined as the blockage of vascular supply to a tumor. The blockage is usually performed via an endovascular approach but may also be performed by direct percutaneous injection of embolic agents into the tumor. For preoperative evaluation of patients, angiography remains the most accurate method for the diagnosis of liver tumors. In the early 1970's, angiographers were becoming more therapy oriented and began to use embolization agents in some angiographic procedures to treat liver tumors for palliative effects [18]. In 1974, Doyon et al reported in French embolization of hepatic artery to treat malignant liver tumors [19]. After that, some authors reported in Japanese transhepatic catheterization and subsequent embolization, or hepatic artery embolization to treat unresectable HCC [20]. In the late 1970's, intrahepatic arterial injection of separate adriamycin, 5-fluorouracil, mitomycin-C, or their combinations came into use for treatment of HCC [21, 22]. It seemed that even one shot of one of these chemicals by intraarterial infusion therapy was superior to by the method of repeated high-dose systemic administration [23]. These agents were soon applied in procedures of bland hepatic artery embolization in therapy for HCC [24]. In the early 1980s, it evolved with the name transcatheter arterial chemoembolization (TACE) and was clinically applied to various HCCs apart from those with emergencies caused by symptomatic humoral hypercalcemia [25]. 

## 3. TAE versus TACE 

Transcatheter arterial embolization (TAE), known as bland embolization, refers to the embolization of the hepatic artery without using any chemotherapeutic agents [26]. When TAE is combined with prior injection into the hepatic artery of chemotherapeutic agents, namely, the former one-shot arterial chemotherapy is integrated into TAE, the procedure is known as TACE [27, 28].

Embolization as a therapeuticprocedure needs accurate placement of artificial emboli and was first used to treat large cerebral angiomatous malformations for which neither ligation of the great vessels in the neck nor X-ray therapy had been found helpful [7]. Intra-arterial application of an embolizing agent is essential in both TAE and TACE to block the feeding vessels of the tumor and deprive nutrient and oxygen supply to the tumor cells. Embolization also causes tumor cell death and suppresses tumor growth [29]. 

The difference between TAE and TACE is the involvement or noninvolvement of regional injection of chemotherapy drugs into the blood vessels that provide nutrients to the tumor [30–32]. For more than a decade, the argument over the rationale of chemotherapy drugs goes on and on. Those who support TACE propagate that TACE appears to be superior to TAE [33]. On the other hand, a number of investigators suggested that embolization alone gives the same survival advantage and TACE may not be better than TAE [30–32, 34].

TACE derives its beneficial effect by two methods. Since most tumors are supplied by the hepatic artery, arterial embolization interrupts their blood supply and postpones growth until replaced by neovascularity. Secondly, focused administration of chemotherapy allows a higher dose to the tissue while simultaneously reducing systemic exposure, which is typically the dose-limiting factor. With high concentration of drugs in the tumor area, the cytotoxic effect on the tumor cells is enhanced and side effects of the chemotherapy drugs are reduced. This effect is potentiated by the fact that the chemotherapeutic drug is not washed out from the tumor bed after embolization [32].

As a matter of fact, both TAE and TACE create significant objective treatment response evaluated by Response Evaluation Criteria  in Solid Tumors (RECIST criteria). As early as 1998, a randomized, controlled trial concluded that TAE has a marked antitumoral effect associated to a slower growth of the tumor, but it does not improve the survival of patients with nonsurgical HCC [34]. One recent study of TACE treating 25 HCC nodules reported response rate of 48% at 1 month and 40% at 3 months [35]. Arterial blood supply into the hepatic tumor is greatly related to objective treatment responses [36]. A number of studies have shown that TACE is a good choice for treating early—intermediate—and advanced-stage HCC with good hepatic reserve for the result of prolonged overall survival [37–40]. 

The advocators of TACE argue that embolotherapy and regional chemotherapy are synergic since tumor ischemia caused by embolization elevates drug concentration compared to sole infusion and prolongs the time of retention of chemotherapeutic drugs, and with repeated TACE, the lifespan for a patient with unresectable HCC could be rationally extended for 1-2 years even more although the exact benefit depends to a great extent on the patient's illness [41]. In addition, TACE at times presents remarkable survival benefit by its high success rate for hemostasis. A recent study on TACE managing spontaneously ruptured HCC reported that TACE increased the 30-day survival in patients with a ruptured HCC [42]. However, this is not a controlled study and theoretically TAE should have the same or similar survival benefit by the effect of hemostasis. 

On the other hand, those who believe the use of chemotherapeutic drugs is unnecessary suggest that TAE may be as equally effective as TACE for treatment of HCC. Most of them are evidence based. They state that no clinical evidence up to now has demonstrated that TAE is less effective than TACE although there is a trend toward enhancing survival with TACE [31]. A systematic review with the meta-analysis of TACE versus TAE alone (three studies, *n* = 412) demonstrated no survival differences (*P* = 0.052) between the two techniques. The authors also compared several anticancer drugs often used in TACE and concluded that no chemotherapeutic agent seemed better than any other [30]. 

Nevertheless, in recent years, TACE has replaced TAE as the most extensively used and investigated palliative modality for unresectable HCC [43]. The majority of researchers maintain that TACE appears to be superior to TAE. Moreover, neuroendocrine tumors and carcinoids in particular, have a significantly greater partial response rate to TACE although HCC is very chemoresistant and embolization is more important than chemotherapy [33].

## 4. TACE Today

Until now TACE has made a main method in the therapy of liver tumors and is considered the gold standard for treating intermediate-stage HCC [44]. Today sole use of TACE as a locoregional therapy gains a complete local tumor control of 25–35% and permits an increase of survival in patients with intermediate HCC according to Barcelona-Clinic Liver Cancer (BCLC) classification [41]. TACE is currently mostly taken as a regional treatment of inoperable HCC, but more and more studies concluded that it is an alternative to resectable early-stage HCC [38, 40]. It is also indicated for patients with regional recurrence in the liver after previous resection of HCC [45]. Another important use of TACE is to downstage HCC in patients who exceed the Milan criteria for liver transplantation. In other words, TACE makes the tumors shrink enough to lower the stage of the cancer. Selected patients with stage III/IV HCC can be downstaged to Milan criteria with TACE, and in patients with a complete response to TACE, excellent posttransplantation outcomes are expected with great disease-free and overall survival [46, 47]. 

Today TACE falls into the category of minimally invasive image-guided therapies for HCC [48]. It is performed via a percutaneous transarterial approach in an angiography apartment by an interventional radiologist or a gastroenterologist. Laboratory studies are completed before the procedure, including liver function, blood count, and the coagulation profile. The patient is abstained from food for four hours. Prophylactic antibiotics are usually not given before and after the procedure. A written consent is obtained after the nature and purpose of the TACE procedure are fully explained. Under local anesthesia, usually the common femoral artery in the right groin is punctured. By Seldinger's technique, the doctor places an arterial sheath in the artery and inserts a catheter through the sheath. Then the catheter is manipulated, sometimes guided by a wire, through the abdominal aorta into the celiac trunk under imaging guidance. During the procedure, contrast medium is occasionally injected to view the arteries and to observe the head point of the catheter. A selective angiography of the celiac trunk (in certain cases the superior mesenteric artery) is routinely done to make a more detailed diagnosis which is now the gold standard of diagnosis of the disease and often overcomes certain diagnostic deficiencies arised from other imaging modalities. Then the catheter is passed through the common hepatic artery, into the proper hepatic artery to reach the target branches that are supplying blood to the tumor. Once the feeding branch is found, the doctor will inject 5 to 10 mL of iodized oil mixed with chemotherapeutic agent and then the embolization particles. After the procedure, the doctor removes the catheter and sheath and applies pressure to the entry site for 5 to 20 minutes to prevent bleeding. The patient remains on bed rest overnight and is discharged the next day. If complications occur, the patient must be kept in hospital for several days to manage them. 

## 5. Role in Comprehensive Treatment of HCC

### 5.1. Effects and Technique Improvement

TACE is palliative or curative for the treatment of HCC [45]. Repeated TACE improves the overall survival, but the most appropriate interval of repetition is uncertain. TACE treatment is repeated every two to three months by many authors [49]. There is no standardized protocol about the choice, dosage, concentration, rate of injection of the chemotherapeutic agent, and optimal retreatment strategy. Also, there is no standard choice for either the embolizing agent or its volume to be used [50, 51]. The number of treatment sessions depends on the response of the tumor and whether serious side effects are seen [30, 50]. The overall response rate of the tumor to this treatment is about 50%, reported with the lowest around 15% and the highest around 85% [33, 35]. Serum albumin level, Child-Pugh class, tumor number, size, alpha-fetoprotein, alanine aminotransferase, des-gamma carboxy-prothrombin, and gamma-glutamyl transferase are correlated to treatment outcomes [52, 53]. Fewer tumor numbers, smaller tumor size, and better liver function predict better tumor response and higher survival rates. Complete disappearance of the tumor shadow is uncommon, but complete tumor radiological response provides a good chance for surgery in patients initially diagnosed with unresectable HCC [54]. 

Different indications of TACE result in different treatment outcomes. By the BCLC staging system, the indication for TACE is multinodular tumors. Recently, an indication in Japan for HCC makes difference. This indication recommends TACE for HCC patients with two or three tumors larger than 3 cm or more than 3 tumors. A study following this guideline reported that the overall median and 5-year survivals were 3.3 years and 34%, respectively. And, in patients with two or three tumors larger than 3 cm and Child-Pugh A liver function, the 3-year survival was 55% [50, 55]. It sounds that updating guidelines make sense.

 In recent years, efforts have been directed to improve the delivery system of chemotherapeutic and radiotherapeutic agents to be used in TACE [27, 40]. Drug-eluting particles are designed to localize a drug to the targeted tumor and minimize systemic exposure to the drug, thus decreasing the common postoperative adverse effects associated with chemoembolization with lipiodol. There are two different types of drug-eluting particles: polyvinyl alcohol microspheres and superabsorbent polymer microspheres. These particles release the loaded drug locally in a slow and sustained manner and cause tumor ischemia by their embolization effect [56–58]. The drug loaded in microspheres is mostly doxorubicin, and other products such as gelatin microspheres loaded with cisplatin have also been reported [35]. Radiotherapeutic agents used in TACE include yttrium-90 microspheres, iodine-131 lipiodol, rhenium-188 lipiodol, phosphorus-32 glass microspheres, and holmium-166 chitosan complex [59]. However, results are conflicting. Some investigations reportedly concluded that these systems might improve the effect on tumor necrosis, with advantage in terms of overall survival and objective complete responses in favor of TACE with these new agents for patients with unresectable HCC. Others demonstrated that, compared with TACE using lipiodol, TACE with calibrated drug-eluting beads loaded with doxorubicin had a similar tumor response and radioembolization with yttrium-90 microspheres had a similar median survival [50]. In addition, one study from a single center showed that lipiodol TACE was not inferior to TACE with drug-eluting beads in terms of response rate and was superior to the latter regarding time to progression. What is more, this study demonstrated that the median overall survival was 46 months for lipiodol TACE and only 19 months for TACE with drug-eluting beads [58]. 

### 5.2. TACE and Liver Surgery

In the past, surgeons have already realized that treatment of HCC by surgery alone has limitations for prolongation of life and multidisciplinary treatment is necessary. Since 1983, some surgeons began to use the catheter treatment to prevent recurrence by embolization with lipiodol-adriamycin mixture followed by gelfoam cubes. The treatment is performed as a rule one month after HCC surgery at three-month intervals for one year [60]. Postoperative TACE proves to reduce intrahepatic recurrence and has been shown with both disease-free survival and overall survival benefits in some papers; however, it was also questioned by others [61, 62]. Although preoperative TACE was evaluated as ineffective on prolonging survivals, TACE has long been used to downstage HCC for liver surgery. In some patients with critically inoperable tumor, the tumor size is reduced after repeated sessions of TACE and the tumor thus becomes resectable [63]. Repeated TACE is regarded as an alternative nonsurgical approach, for post-TACE resection may prolong the survival time before liver transplantation can be performed, thus significantly prolonging the survival rate of patients with HCCs. Issues are paradoxical and even controversial, in favor of post-TACE resection and unfavorable for preresection TACE, but it is the same thing for TACE itself. 

Things are different now. In the late 1990s, TACE has been used as an alternative for resectable HCC [38]. A recent study from Taiwan compared the long-term outcome of TACE with liver resection. All the included patients had resectable early-stage HCC and Child-Pugh class A liver function. The mean survival times of both groups are similar. The 1-, 3-, and 5-year overall survival rates of the TACE group and the liver resection group were 91%, 66%, and 52% and 93%, 71%, and 57%, respectively. This study confirmed that TACE is efficient and safe for resectable early-stage HCC with overall survival rates similar to that of liver resection and is indicated in selected patients with resectable early-stage HCC [40]. Besides, TACE has several benefits over surgical resection including the anticipated reduction in morbidity and mortality, minimal trauma and pain, short hospital stay and recovery, low cost, suitability for real-time image guidance, and cosmesis [48].

### 5.3. Combined Use of TACE with Other Local Ablative Approaches

The combination of TACE with other local ablative therapies has achieved excellent results in the treatment of HCC. For example, overall survival is improved when it is combined with percutaneous ethanol injection (PEI) or radiofrequency ablation (RFA), as neoadjuvant therapy prior to liver resection, or as a bridging tool before liver transplantation [41].

TACE alone is rarely able to produce a full necrosis of large lesions. PEI is often unable to produce homogeneous distribution of ethanol within a tumor. TACE causes disruption of internal septation and decrease of density within a tumor, as well as a fibrous peritumoral reaction. These changes facilitate the use of TACE followed by PEI, with the injected ethanol spreading more evenly and confined better within the tumor. HCC patients with large or multiple tumors treated by the combination of TACE and PEI have a clear survival benefit with muchbetter survival rates at one, three, and five years [64].

 For HCCs with multiplicity, RFA has its limitations in terms of the size and number of HCCs that can be treated. And some locations in the liver might be difficult to approach even with imaging guidance. Several studies have confirmed that the combination of TACE and RFA yields better local tumor control and decreases tumor recurrence compared with RFA alone in the treatment of patients with HCC [65, 66]. In addition, before the performance of RFA, a TACE in the same session is helpful for detection of multiplicity and for localization of the tumor by the deposit of lipiodol. 

Conventional radiotherapy presents limited efficacy for the treatment of HCC and the patient often cannot tolerate this therapy especially when liver cirrhosis exists. Stereotactic body radiation therapy delivers a high dose in a short time to the well-defined target area, with rapid dose fall-off gradients, thus reduces liver toxicity. It has been used as a bridge therapy for patients with HCC awaiting liver transplantation and its combination with HCC seems promising to improve treatment efficacy [67, 68]. When TACE is used in combination with three-dimensional conformal radiotherapy, 1- and 2-year survival rates have been reported to be similar to that of surgery in a cohort of HCC patients with portal vein thrombus [69].

## 6. Prospects

The debate on the problem of TAE versus TACE will continue. HCC is resistant to chemotherapy, so the chemotherapeutics used in TACE should be retained in the tumor region for the expectation of high intratumoral concentration of the drug. Chemotherapy agents may prevent remote metastasis, but they also may agitate cancer cells to flee. The authors had long-term surviving HCC patients with metastasis in subcutaneous tissue of the abdomen, the back, groin and in the brain, most in the lungs, or vertebrae. These facts make us confused about the effects of chemotherapy agents. At the present, TAE is used when chemotherapy is riskful according to the patients condition. Large cohort studies are necessary to further define various TACE protocols for improving therapeutic outcome of HCC, especially in terms of patient population selection. Necessity and timing of repeated embolization will be further investigated. After protocol modification, some treatment strategies with TACE will become superior from potentially curative to curative methods for the treatment of HCC. 

 Embolization makes ischemia of the affected tissues. TACE-generated ischemia can raise angiogenesis factors which accelerate regeneration of residual tumor cells and account for the high rate of local recurrence. The combination use of antiangiogenic drugs and TACE should help to decrease the recurrence rate, but this must be verified in clinical studies. Sorafenib in combination with TACE will significantly prolong the life expectancy of HCC patients, although systemic chemotherapies have proved disappointing for the treatment of HCC [29]. After using such angiogenesis inhibitors, antiangiogenesis resistance may develop in HCC. Novel agents are being investigated to overcome this resistance, such as brivanib [70]. Several other angiogenesis inhibitors are also in development to treat HCC both for first-line use and for use following sorafenib failure [70]. 

Nowadays, TACE as a local ablative treatment is able to induce local disease control and to prolong survival and might even achieve survival similar to surgical resection; however, the high rates of recurrence of HCC after successful control of local tumor spread are the reason to consider that procedure as a noncurative treatment option. In contrast to necrosis resulted from TACE, apoptosis is not commonly accompanied by an inflammatory response that causes collateral cell damage. Thus combination of intratumoral or intraarterial injection of p53 products with TACE, with embolization effects of tumor tissue ischemia and necrosis, may be synergic and improve survival. It is an attempt to achieve a synergism of external stimuli and cellular p53 gene expression for the induction of apoptosis of liver cancer cells. 

 For improving the performance of TACE and reducing TACE practitioner's exposure to X-ray, we now have the concept of robot-assisted catheter insertion. Inspired by the idea of computer-aided surgery and taking advantage of the rapid advancement in imaging and robotic technologies, researchers will construct an integrated performance navigation and medical robotic system for safer and more refined practice of catheterization [71]. 

## 7. Conclusion

TACE develops gradually from the procedures of diagnostic angiography and transcatheter injection of agents and is in particular performed in the treatment of HCC. TACE also has a role in delaying the progression of HCC as a bridge therapy to orthotopic liver transplantation. In recent years, it is indicated in selected patients with early-stage HCC. Careful patient selection is crucial for great outcome in the treatment of HCC with TACE. Fewer tumor numbers, smaller tumor size, and better liver function predict better tumor response and higher survival rates. Current protocols of TACE are heterogeneous in the choice, dosage, concentration, rate of injection of the chemotherapeutic agent, and optimal retreatment strategy, usually decided by institutional and personal referral. Large cohort studies are necessary to further define various TACE protocols for improving therapeutic outcome of HCC. Combination use of TACE with other local ablative therapies has achieved excellent results in the treatment of HCC.
